# Refractive change in the adult rabbit eye after corneal relaxation with the femtosecond laser

**DOI:** 10.1186/1471-2415-14-8

**Published:** 2014-01-21

**Authors:** Zhen-Yong Zhang, Matthew R Hoffman, Xing-Tao Zhou, Ye Xu, Xing-Ru Zhang, Ren-Yuan Chu, Chong-Da Chen

**Affiliations:** 1 Department of Ophthalmology, Putuo Hospital, Shanghai University of Chinese Traditional Medicine, No. 164, Lanxi Road, Shanghai (200062), China; 2 Department of Surgery, University of Wisconsin School of Medicine and Public Health, Madison, WI, USA; 3 Department of Ophthalmology, Eye & ENT Hospital, Fudan University, 19 Baoqing Road, Shanghai 200031, China; 4 Department of Ophthalmology, The Affiliated Cixi Hospital of Wenzhou Medical University, Cixi, China

## Abstract

**Background:**

A new procedure to correct myopia that does not disturb the cornea in the optical zone and avoids injuring the corneal epithelium could be a key advance in corneal refractive surgery. The aim of this study is to observe the refractive change in the adult rabbits undergoing femtosecond laser-assisted multilayer intrastromal ablation in the mid-periphery of the cornea without injury of epithelium.

**Method:**

The right eyes of 8 New Zealand White adult rabbits were used for the experiments. A 60-kHz femtosecond laser delivery system was used, and three lamellar layers of laser pulses were focused starting at a corneal depth of 180 μm and ending at 90 μm from the surface, with each successive layer placed 45 μm anterior to the previous layer. In the interface of the applanation contact lens cone, a 6-mm diameter aluminum circle was placed at the center to block the laser, limiting ablation to the mid-periphery of the cornea. The laser settings were as follows: spot/line separation, 10 μm; diameter, 8.0 mm; energy for ablating the stroma, 1.3 μJ. An authorefractor was used to assess the manifest refraction.

**Results:**

Mean spherical equivalent (SE) (mean ± SD, SD: standard deviation) was significantly increased at postoperative week 1 (1.67 ± 0.26 D, p < 0.0001), month 1 (1.65 ± 0.23 D, p < 0.0001), and month 3 (1.60 ± 0.22 D, p < 0.0001) compared to baseline (0.68 ± 0.27 D). Mean spherical equivalent showed no significant change between postoperative week 1 and month 3 (p = 0.1168).

**Conclusion:**

Femtosecond laser-assisted multilayer corneal intrastromal ablation in the mid-periphery may cause a consequent hyperopic shift with no refractive regression.

## Background

The cornea, the most important refractive element of the eye, supplies two thirds of the total refractive power and makes it an appealing target for most refractive surgical procedures, of which the ablative procedures such as photorefractive keratectomy (PRK), laser in situ keratomileusis (LASIK) and laser epithelial keratomileusis (LASEK) are based on corneal tissue removal, whereas the incisional procedures, such as radial keratotomy (RK), correct myopia by relaxing the corneal tissue. The ablative procedures are to ablate a cornea in the optical zone and thus carry risks of central cornea scarring as a result of unfavorable wound healing. RK has the advantage of sparing treatment over the visual axis and not disturbing the cornea in the optical zone; however, corneal incisions result in keratocytes differentiating into myofibroblasts which play a role in the development and subsequent contraction of scars at the sites of incisions. And, it is accepted that preserving the epithelium is a key factor in wound healing and avoidance of postoperative complications [1]. Accordingly, a new procedure to correct myopia that does not disturb the cornea in the optical zone and avoids injuring the corneal epithelium could be a key advance in corneal refractive surgery.

In 2000, the femtoseond laser (FSL) was approved by the Food and Drug Administration and was thereafter extensively used to make a corneal flap for LASIK; complications during LASIK related to the flap were therefore significantly reduced [2-4]. Then, too, FSL makes it possible to relax a cornea by ablating the mid-peripheral stroma without injuring the epithelium, with a potential refractive change occurring due to intraocular pressure. In a previous study [5], we demonstrated the morphological and histopathologic changes to the immature rabbit cornea after multi-layer ablation of the stroma with the FSL at different depths in the mid-periphery of the cornea, which may not have been definitive because of immaturity of the cornea. In this study, we present the refractive change in the adult rabbits undergoing this procedure.

## Methods

### Animals and examination procedure

The right eyes of eight New Zealand white adult rabbits weighing 2.5 kg to 3.0 kg were used for the experiments. All animals used in this study were treated according to the guidelines of the Association for Research in Vision and Ophthalmology. And this study was approved by the Ethics Committee of the Eye & ENT Hospital, Fudan University, Shanghai, China. Prior to the experiments, the animals were examined with a slit lamp to rule out clinically observable ocular diseases. The manifest refraction was determined using the autorefractor (ARK-700A; NIDEK Co., Ltd., Aichi, Japan) and recorded as spherical equivalent (SE). Corneal power was determined using a corneal topography system (ZEISS, Model 995).

Follow-up examinations were performed with topical anesthesia at postoperative 7, 30, and 180 days.

### Femtosecond laser procedure

**Figure 1:**
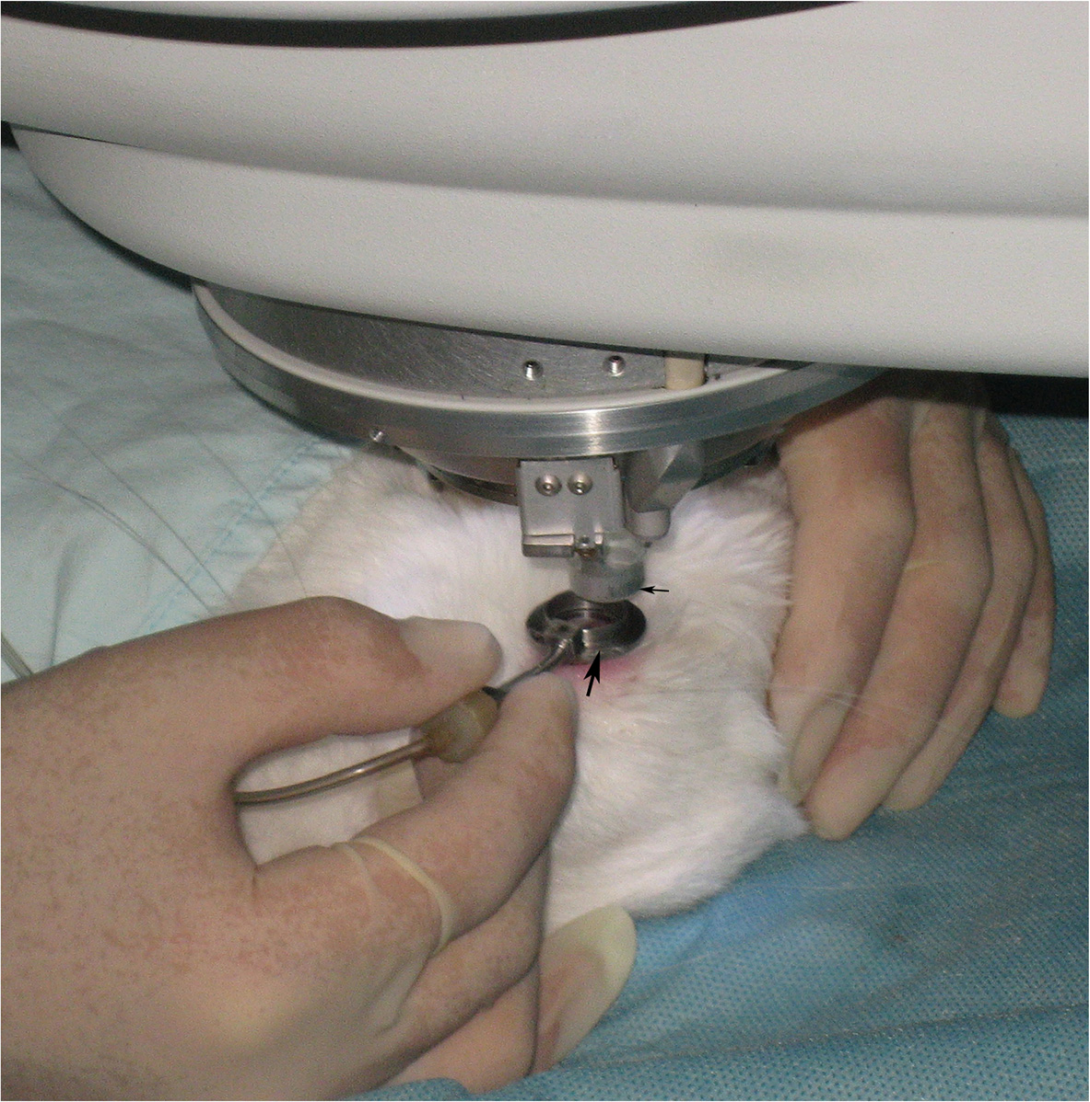
The rabbit eye was fixed with a special suction ring (big arrow) and an applanating contact lens (little arrow) was being docked into the suction ring.

**Figure 2:**
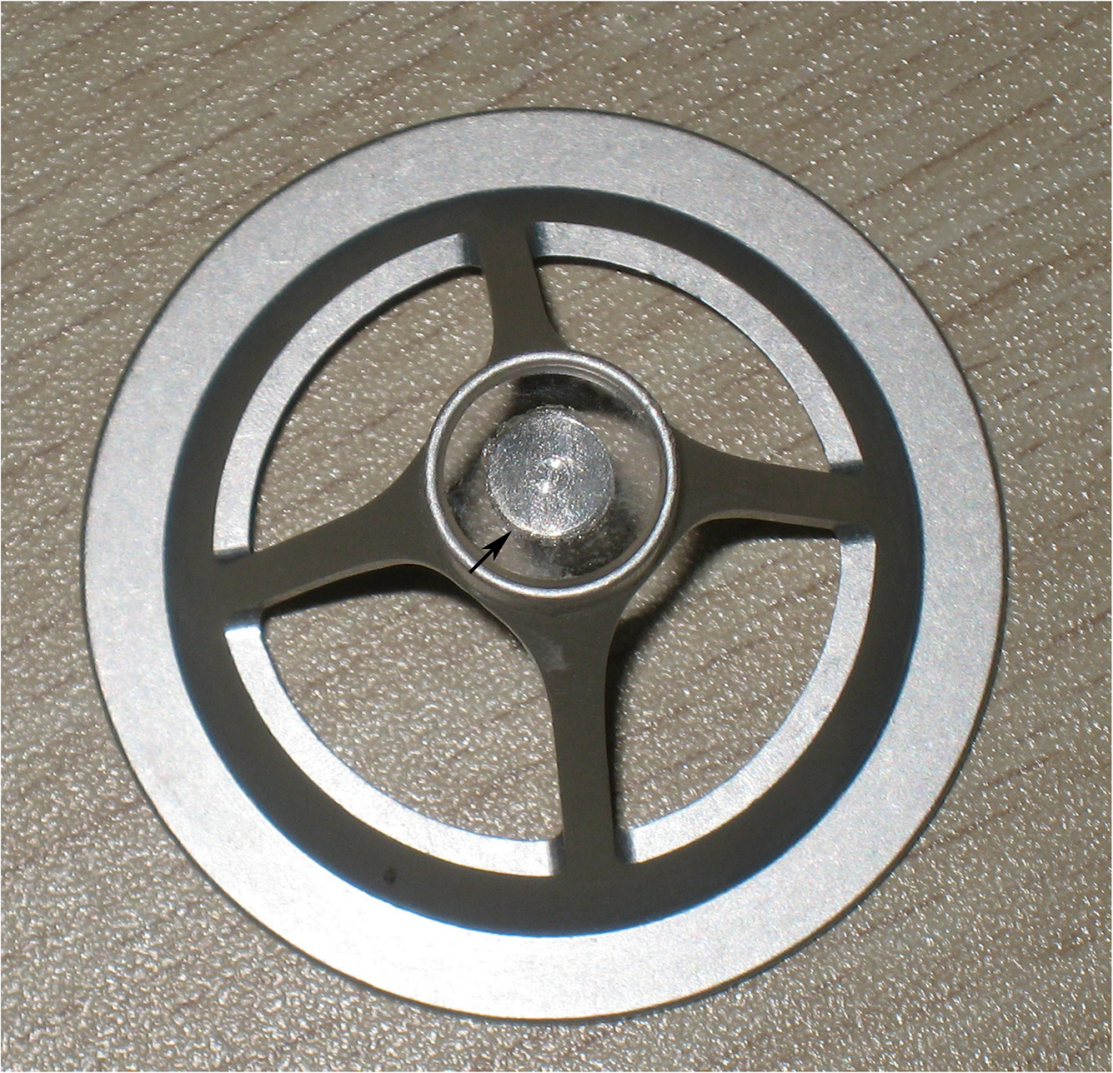
The contact lens with a 6-mm diameter aluminum circle at its center (arrow).

Animals were premedicated intramuscularly with an injection of diazepam (1 mg). For general anesthesia, 10% ketamine hydrochloride (35 mg kg^-1^ of body weight) was injected intramuscularly. For additional local anesthesia, 0.5% dicaine eye drops were applied to the right eyes. Eyes were fixed with a special suction ring which was connected with the Moria 2 microkeratome system used to produce suction pressure (Figure 1). The cornea was applanated with the disposable applanating contact lens cone located at the tip of the 60 kHz femtosecond laser (Intralase FS, Advanced Medical Optics, Irvine, CA) delivery system. A 6-mm diameter aluminum circle was placed at the center of the interface of the lens cone to block the laser (Figure 2). This design ensured that only the corneal stroma in the mid-periphery was ablated by the laser. For intrastromal ablation, three lamellar layers of laser pulses were focused starting at a corneal depth of 180 μm and ending 90 μm from the surface with each successive layer placed 45 μm anterior to the previous layer. No edge cuts were performed. The laser settings were: spot/line separation, 10 μm; diameter, 8.0 mm; energy for ablating the stroma, 1.3 μJ.

### Statistical analysis

All statistical analyses were performed using the Stata10.0 statistics program (Stata Corp, Texas). One-way repeated measures analysis of variance (ANOVA) was performed followed by paired t-tests. A significance level of α = 0.05 was used for all tests.

## Results

**Figure 3:**
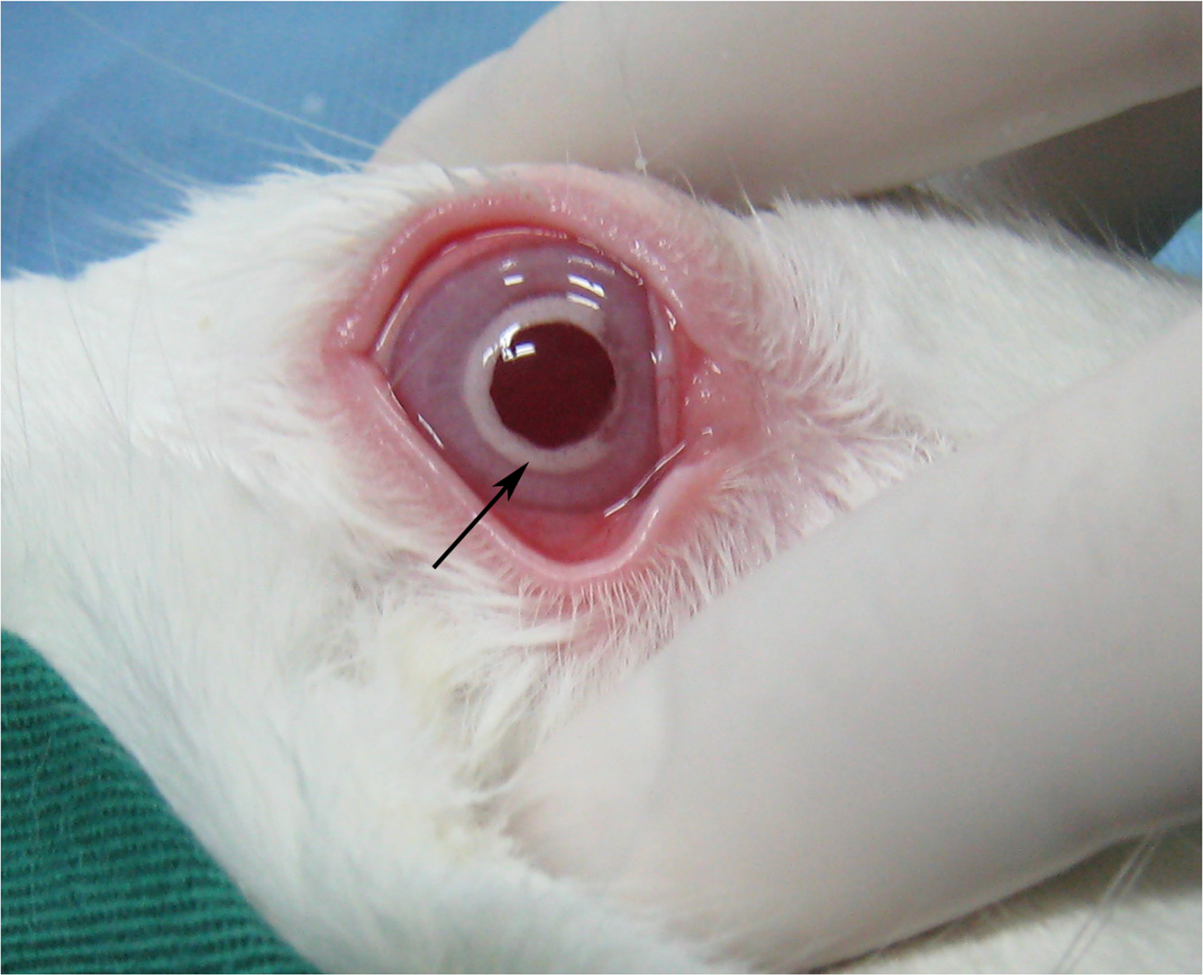
**The bubbles appeared within an area of 6 to 8.0 mm in the corneal stroma (*arrow*).**

**Figure 4:**
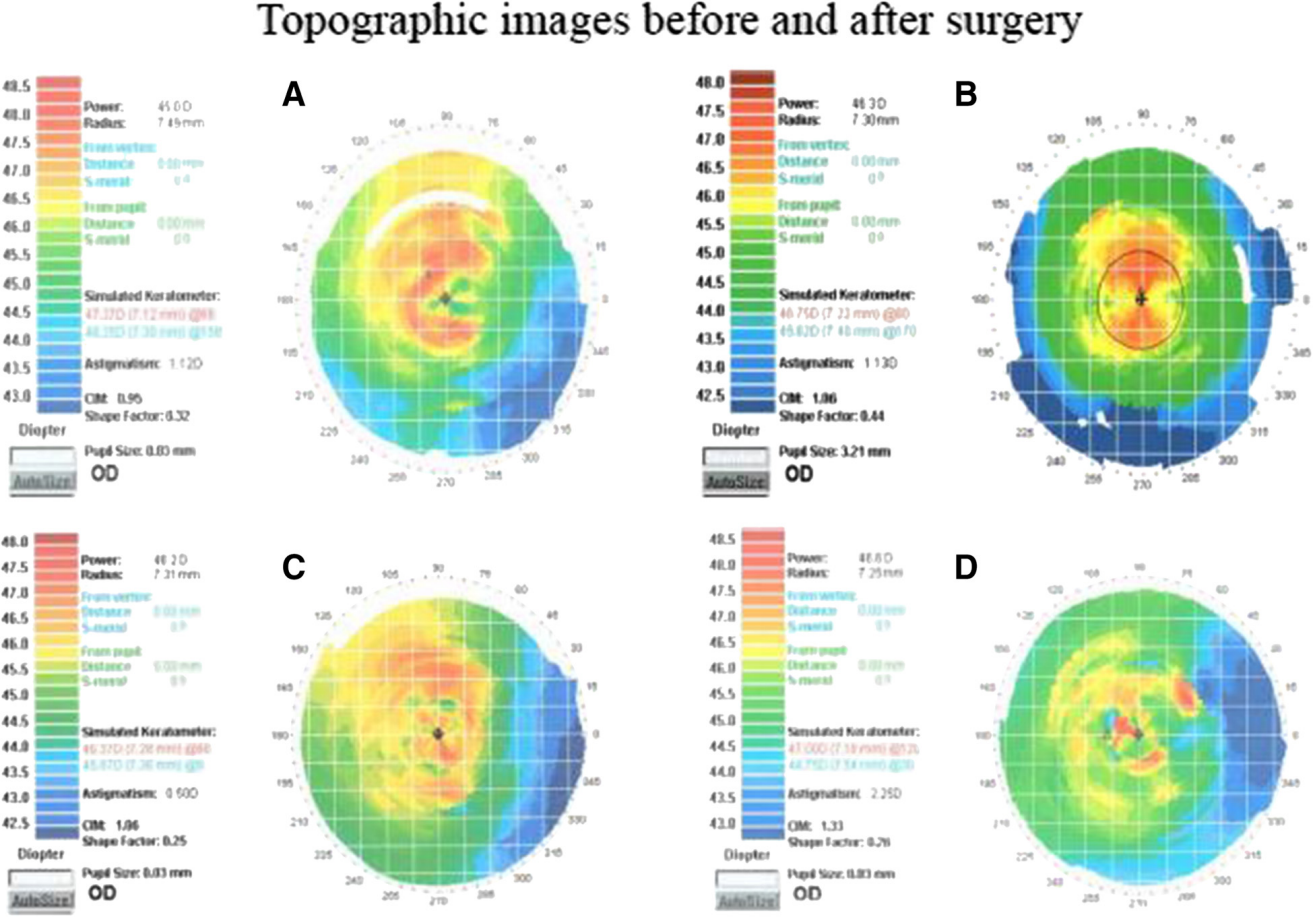
**Images of the rabbit corneal topography before surgery (A) and on postoperative days 7 (B), 30 (C), and 90 (D).** These images indicated that mean corneal power decreased postoperatively.

**Figure 5:**
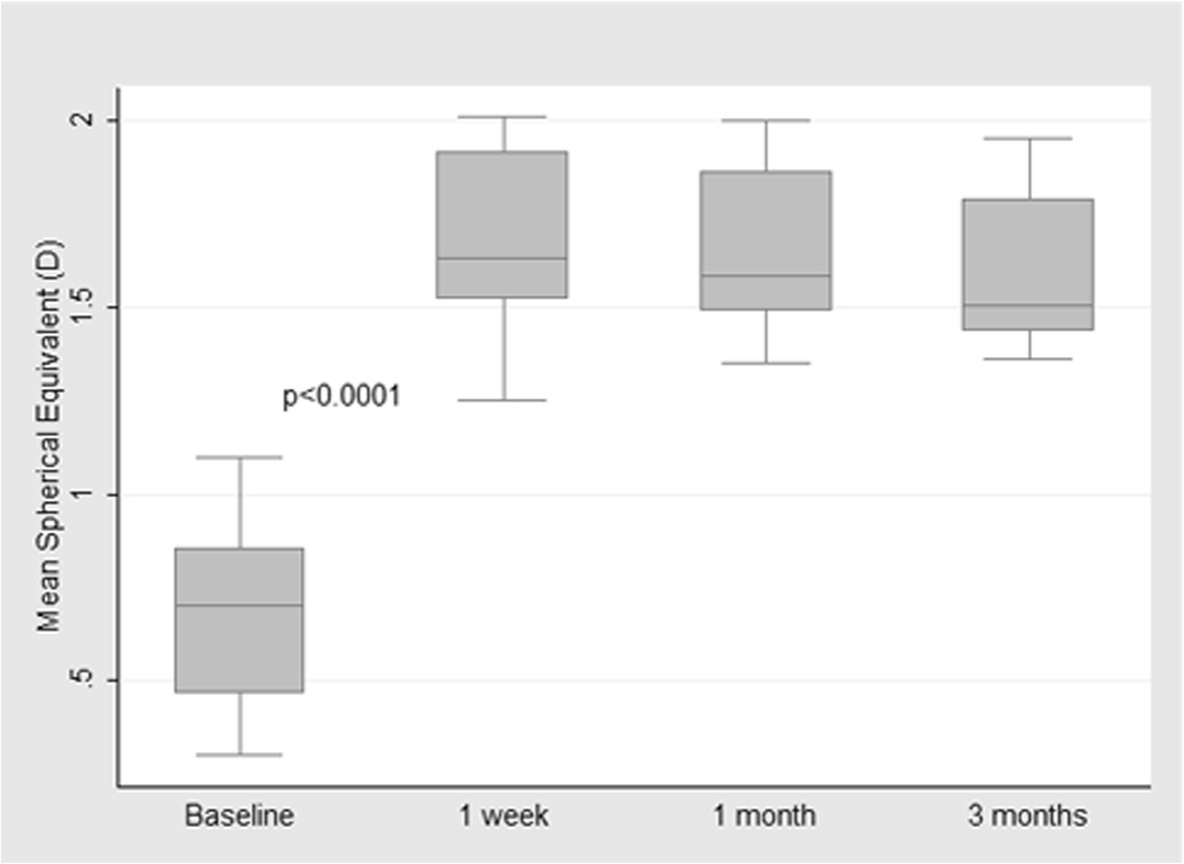
**Mean spherical equivalent at baseline and at indicated times after surgery.** There was a significant difference between baseline and postoperative week 1 (p < 0.0001), whereas no significant change was found between postoperative week 1 and month 3 (p = 0.1168).

Microbubbles accompanied each lamellar layer ablation and appeared within the scope of 6 to 8.0 mm in the corneal stroma after surgery (Figure 3). These microbubbles would last about 30 minutes, after which the cornea became transparent again. Mean manifest refraction (mean ± SD, SD: standard deviation) was significantly increased at postoperative week 1 (1.67 ± 0.26 D; t = 12.5595, p < 0.0001), month 1 (1.65 ± 0.23 D; t = 14.0381, p < 0.0001), and month 3 (1.60 ± 0.22 D; t = 13.8982, p < 0.0001) compared to baseline (0.68 ± 0.27 D) (n = 8) (Figures 4A-D). Mean manifest refraction showed no significant change between postoperative week 1 and month 3 (t = 1.7885, p = 0.1168) (n = 8) (Table 1, Figure 5).

**Table 1:** Mean spherical equivalent before and after surgery

	**Mean spherical equivalent (D)**			
**Rabbit**	**Baseline**	**1 week**	**1 month**	**3 months**
1	0.81	2.01	2	1.95
2	0.78	1.91	1.89	1.8
3	0.9	1.5	1.61	1.52
4	0.5	1.6	1.56	1.49
5	1.1	1.92	1.83	1.78
6	0.43	1.25	1.35	1.42
7	0.62	1.66	1.48	1.45
8	0.3	1.54	1.5	1.36

## Discussion

The FSL is a mode-locked, diode-pumped, neodymium/glass laser that produces near-infrared pulses and cannot be absorbed by optically clear tissue [6]. The laser, therefore, can be focused anywhere within the cornea where the energy can be raised to a threshold such that small volumes of corneal tissue are vaporized and plasma, a shockwave, cavitation, and gas (CO_2_ and H_2_O) bubbles are generated [7]. This laser-induced optical breakdown, also called photodisruption, can act as a nonthermal ablative process which allows the stromal layers to be divided and a LASIK flap to be created. It has been shown in a previous study that corneal tissue is removed because of the effect of the laser plasma and the most efficient tissue removal can be achieved by placing the approximately spherical microbubbles adjacent to each other [8]. To that end, appropriate laser parameters should be applied for an efficient dissection of the corneal stroma. For the 60 kHz FSL, a spot/line separation as low as 4 × 4 μm and pulse energy less than 1 μJ can be used to create a superior stroma bed [7]. If a larger spot/line separation is set, the laser may induce corneal relaxation rather than corneal dissection by the disruption of integrity of the cornea due to the consequent unconnected microcavitations in the stroma. In this study, intrastromal ablation was performed in the mid-periphery of the anterior cornea with a 10-μm spot/line and 45-μm layer separation whereby the unconnected microbubbles were generated between and among the layers. And it is believed that the greatest strength of the cornea lies within the anterior stroma and in the periphery [9], where the lamellae are more tightly packed. With this understanding, these ablations may result in the midperipheral corneal relaxation and subsequent corneal flattening due to intraocular pressure; this can explain the finding of an approximate 1.0 D hyperopic shift in the present study, which was stable up to postoperative 3 months. Nevertheless, this is inconsistent with our previous study in which immature rabbits were used and the resulting corneal power from a similar procedure was unstable with an additional decrease of approximately 1.0 D from postoperative 1 month to 3 months [5]. This disparity may lie in the difference in the age of the included rabbits as immature rabbit has been reported to experience corneal flattening while maturing [10]. Of note, this discrepancy may be complicated by thickening of the lens of the immature rabbit eye [11]. With these understandings, the present study that has investigated the change in refraction rather than in corneal power in the adult rabbit cornea is arguably superior in methodology to the previous study and thus yields a stronger conclusion, which can in part be supported by the finding of a myopia correction of 1.12 D in a human eye that underwent a similar surgery in another study [12].

Because clinical outcome depends partly on the corneal repair response following the procedure, it is important to consider how the cornea would heal after intrastromal ablation. In a study on isolated stromal injury using the FSL, Meltendorf et al. [13] demonstrated that no keratocytes differentiated into α-smooth muscle actin (α-SMA) positive fibroblasts, and that transforming growth factor β_1_ (TGF-β_1_) expression did not significantly increase [14]. α-SMA is a myofibroblast marker [15] and myofibroblasts appear to be responsible for the formation of haze [16]. In addition, regression after corneal refractive surgery is attributable to epithelial hyperplasia and stromal remodeling [17,18], both of which involve TGF-β [19]. These findings not only lend further support to what we observed in this study in which no refractive regression occurred during follow-up, but also make us believe that the wound healing process after this procedure is different than the one observed with current ablative procedures. However, longer follow-up is needed to rule out late-onset refractive regression.

However uncertain the improvement of this procedure over currently used surgical techniques, a procedure that corrects myopia avoiding injury to the corneal epithelium and sparing the optical zone may represent an important advance in corneal refractive surgery. Further studies determining optimal laser settings, layer separations, optical zone, ablation layers, and beginning and ending ablation depths are warranted. This would allow for procedural optimization and also standardization of which settings should be applied to correct different degrees of myopia. If such an algorithm were developed and long-term post-operative healing was without complications, consideration of this procedure as an alternative to LASIK and LASEK would be merited.

## Conclusions

Femtosecond laser-assisted multilayer intrastromal ablation in the mid-peripheral cornea may induce a mild shift of refraction in hyperopic direction with no refractive regression that may point to a wound healing mechanism having yet to be elucidated.

## Abbreviations

PRK: Photorefractive keratectomy; LASIK: Laser in situ keratomileusis; LASEK: Laser epithelial keratomileusis; RK: Radial keratotomy; SE: Spherical equivalent; FSL: Femtoseond laser.

## Competing interests

The authors declare that they have no competing interest.

## Authors’ contributions

ZZY and CRY conceived the study; ZXT, ZZY and XY performed all experiments; ZZY wrote and revised the manuscript; HMR and CCD interpreted the data and revised the manuscript; ZXR supervised the study. All authors read and approved the final manuscript.

## Pre-publication history

The pre-publication history for this paper can be accessed here:

http://www.biomedcentral.com/1471-2415/14/8/prepub
